# From incoherence to mirth: neuro-cognitive processing of garden-path jokes

**DOI:** 10.3389/fpsyg.2015.00550

**Published:** 2015-05-12

**Authors:** Bastian Mayerhofer, Annekathrin Schacht

**Affiliations:** Courant Research Centre “Text Structures,” University of GöttingenGöttingen, Germany

**Keywords:** jokes, verbal humor, semantic revision, discourse comprehension, event-related potentials, N400, P600, pupil diameter

## Abstract

In so-called garden-path jokes, an initial semantic representation is violated, and semantic revision reestablishes a coherent representation. 48 jokes were manipulated in three conditions: (i) a coherent ending, (ii) a joke ending, and (iii) a discourse-incoherent ending. A reading times study (*N* = 24) and three studies with recordings of ERP and pupil changes (*N* = 21, 24, and 24, respectively) supported the hypothesized cognitive processes. Jokes showed increased reading times of the final word compared to coherent endings. ERP data mainly indicated semantic integration difficulties (N400). Larger pupil diameters to joke endings presumably reflect emotional responses. ERP evidence for increased discourse processing efforts and emotional responses, as assumed to be reflected in modulations of the late left anterior negativity (LLAN) and in an enhanced late frontal positivity (fP600), respectively, remains however incomplete. Processing of incoherent endings was also accompanied by increased reading times, a stronger and sustained N400, and context-sensitive P600 effects. Together, these findings provide evidence for a sequential, non-monotonic, and incremental discourse comprehension of garden-path jokes.

## Introduction

A comedian enters the stage and announces to the audience: “I want to die peacefully in my sleep like my grandfather. Not screaming in terror like his passengers^1^.” One does not need to be a comedian to know this specific moment between the delivery of a joke and the mirthful reaction that hopefully follows it. There is this confused look in the faces that instantly changes to smile and laughter, once the joke is successfully comprehended. But what exactly is happening in the recipient's mind in this very moment between confusion and laughter? Investigating the underlying neuro-cognitive and emotional processes of this very moment can reveal important insights for at least two overlapping research fields: psychology of humor (Martin, 2007), and discourse comprehension (e.g., Kintsch, 1998).

The most important theories of humor in Cognitive Sciences are variations of incongruity(-resolution) theories (Suls, 1972; Nerhardt, 1977; McGhee, 1979; Giora, 1991; Forabosco, 1992). Incongruity is defined as the violation of expectations during the perception and interpretation of a specific situation or communication (e.g., McGhee, 1979, p. 6/7). Much of linguistic and psycho-linguistic research on verbally expressed humor is focused on a specific kind of canned joke which forms a large and fairly homogeneous subclass of verbal humor, even though it rarely is explicitly mentioned as a specific subclass. The joke in the first paragraph serves as an example. This subclass of verbal humor can be called garden path (GP) jokes (Dynel, 2009) or forced re-interpretation jokes (Ritchie, 2004). GP jokes can be described in agreement with incongruity(-resolution) theories. GP jokes are usually rather short humorous texts. They include an ambiguous initial set-up, which allows (at least) two contrastive interpretations. This ambiguity can appear at several linguistic levels (lexical, syntactic, semantic, pragmatic, contextual; see Dynel, 2009, for a detailed analysis of different types of ambiguity in GP jokes); however, it should initially remain undetected by the recipient. The punch-line, usually the ending, violates the automatic and initially dominant or salient (Giora, 2003) interpretation of the ambiguous set-up. This violation triggers incoherence, leading to the perception of incongruity^2^. In order to resolve this incongruity, the recipient needs to re-analyze the meaning of the text and to find an alternative interpretation which is consistent with the new linguistic input provided by the punch-line. The alternative interpretation then re-establishes a coherent meaning of the text.

Contrary to so-called GP sentences (Osterhout and Holcomb, 1992; Ferreira et al., 2001), the violation and the re-analysis of GP jokes are localized at the semantic rather than syntactic level although it is difficult—and a matter of theoretical conceptualization—to clearly disentangle these two levels. Semantic garden-path mechanisms have also been studied in the context of lexical ambiguity (resolution), polysemy and homonymy (e.g., Meyer and Federmeier, 2008). However, in GP jokes, crucially, the mental representation of the discourse, theoretically depicted as mental model (Johnson-Laird, 1983) or situation model (Kintsch, 1988, 1998), is violated at the punch-line. It is commonly assumed that the discourse comprehension is an active process of cognitive construction that involves the integration of explicit linguistic input with other linguistic and non-linguistic context information, including new semantic and pragmatic inferences and knowledge from long-term memory. Most importantly, a committed false belief concerning the interpretation of the text has to be substituted. We propose that this “(belief) revision” of the semantic representation (of the set-up) is the crucial mechanism during the comprehension of GP jokes (cf. Mayerhofer and Schacht, 2013). We will shortly illustrate these processes involved in the following example of GP jokes (1):

(1) –“Mummy, I just turned 14 years. May I please, finally, be allowed to wear a bra and make up.” –“No, you are not. And eat up your soup, my *son*!”

Given the linguistic information and the recipient's world knowledge, the child being a girl is the most plausible interpretation of the set-up phase. This interpretation gets violated when one hears the mother calling the child “son,” thus leading to incongruity. Belief revision occurs, and the recipient represents a boy who would love to wear a bra and make up. This incongruity resolution, in combination with the activation of the alternative, hidden interpretation and with its “inappropriateness” (Ritchie, 2004, p. 61), is typically accompanied by the experience of laughter and mirth in the recipient.

Many researchers agree upon the outlined sequential comprehension process, supported by empirical evidence. Vaid et al. (2003) demonstrated priming effects due to the dominant semantic networks specifically activated at different stages of joke comprehension over time. Coulson and Kutas (1998) found longer reading times for joke endings compared to straight (coherent) endings. These longer reading times were also accompanied by regressive eye movements after reading the punch-line (Coulson et al., 2006). Evidence for the enhanced costs of semantic revision also comes from non-joke texts (Carreiras, 1996; Sturt, 2007). Recently, several studies using event-related brain potentials (ERPs) have investigated the processing of jokes and verbal humor. Three (groups of) ERP components were especially fruitful for the study of verbal humor: the N400, late positivities, and the late left anterior negativity.

The N400 component (Kutas and Hillyard, 1980) is an enhanced negative-going deflection at centro-parietal electrodes starting around 200–250 ms after stimulus onset and lasting until 500–550 ms after stimulus onset with a peak around 400 ms, hence the name. It reliably occurs with semantic violations during sentence or discourse comprehension (Berkum et al., 1999). Other important factors that influence the amplitude of the N400 component are the predictability of a word in a given context, as for example reflected by the Cloze-probability (Kutas and Hillyard, 1984), and the semantic relatedness between the context and the expected word. The N400 effect functionally reflects semantic integration difficulties at the interface of word/stimulus recognition, linguistic, and non-linguistic context, and conceptual binding with the long-term-memory during an active comprehension process (Kutas and Federmeier, 2011). Previous ERP studies on joke comprehension have led to heterogeneous evidence regarding N400 effects. Derks et al. (1997) found augmented N400 amplitudes for jokes that also elicited a higher activation of the zygomatic muscle, indicating the elicitation of positive emotions. Coulson and Kutas (2001) found an N400 effect for joke endings involving frame shifting compared to straight endings. The effect was restricted to jokes with high semantic constraint on the ending. This finding was replicated in follow-up studies, shown to be only present for participants with a low verbal intelligence score (Coulson and Lovett, 2004), and to be related to the visual field of the stimulus presentation (Coulson and Williams, 2005).

Several ERP studies on language comprehension demonstrated syntactic violations to elicit an augmented positivity at posterior scalp sites. This so-called P600 component usually starts around 600 ms after stimulus onset and lasts until around 1200 ms. Since these late positivities are triggered by syntactic anomalies, such as in GP sentences (Bever, 1970; Osterhout and Holcomb, 1992), they are commonly considered to reflect syntactic repair processes which occur after the detection of a syntactic violation. However, Van Herten et al. (2005) found posterior P600 effects for semantic anomalies, and experimentally ruled out the possibility of a hidden syntactic anomaly being responsible for the component. This finding led the authors to argue that the P600 is a form of monitoring component “that checks upon the veridicality of one's analysis” (Van Herten et al., 2005, p. 254). In line with this assumption, the P600 has been suggested to reflect a combinatorial process, integrating both syntactic and semantic features of a sentence (e.g., Wicha et al., 2004; Martín-Loeches et al., 2009), and has also been reported for increased discourse complexity (Burkhardt, 2007). Moreover, a late positivity effect—distinguishable from the typical P600 effect by its frontal distribution—has been reported (Schacht et al., 2010) and related to the complexity and the ambiguity of a text (Kaan and Swaab, 2003). In many regards, GP jokes might be assumed as a semantic equivalent of GP sentences. Thus, the question is whether a semantic repair process in jokes—such as the belief revision—triggers similar brain response patterns as the mainly syntactic repair processes (P600 at posterior sites). Previous evidence has partly indicated such similarity, but remains incomplete (Coulson and Lovett, 2004; Marinkovic et al., 2011).

Apart from the P600 like findings, there is strong evidence that joke endings elicit a left-lateralized sustained anterior negativity (late left anterior negativity; LLAN), between 500 and 900 ms after stimulus onset. This component has been shown mainly for good comprehenders (Coulson and Kutas, 2001; Coulson and Williams, 2005). It was reduced for left-handed participants (Coulson and Lovett, 2004). Coulson and co-workers suggested that the component reflects the successful comprehension of jokes and called this effect “frame-shifting component” according to their conceptual framework. The LLAN has also been considered to reflect working memory activity necessary for the computation of a new mental representation of the discourse (Münte et al., 1998; Baggio et al., 2008; Meltzer and Braun, 2013). Similar activation patterns, most commonly earlier in time though, have been usually reported in studies investigating syntactical violations (see Steinhauer and Drury, 2012). It remains unclear whether these activation patterns that at a first glance appear to be similar, actually reflect a comparable underlying cognitive mechanism. But if they do, on a functional level, these processes are likely to be very broad and general, as for example increased working memory activity.

GP jokes also reliably lead to the subjective experience of mirth. Therefore, one might expect other ERP components elicited by jokes, reflecting the emotional processes. Emotion-related ERP responses to humorous visual stimuli have been reported as Posterior Positivities between 300 and 600 ms after the onset (Gierych et al., 2005; Korb et al., 2012). These components show strong similarities to the late positive complex (LPC), which has repeatedly been shown in response to emotional stimuli, such as affective pictures (e.g., Cuthbert et al., 2000; Schupp et al., 2000), and to facial emotional expressions and emotional words (e.g., Schacht and Sommer, 2009a,b). This effect has been related to sustained, elaborative processing of emotional relevance of a given stimulus. At longer latencies, Du et al. (2012) reported an enhanced positivity to Chinese jokes compared with neutral Chinese texts between 1250 and 1400 ms after the stimulus onset, which the authors related to an affective stage of the joke processing.

It is the main aim of the present study to disentangle different sub-processes or processing stages, respectively, involved in the comprehension of GP jokes to be reflected in distinguishable ERP components over time. At least, three different processing stages are hypothesized to be involved: (a) the violation of the pre-dominant initial semantic representation, (b) the revision of this semantic representation, and (c) the occurrence of an emotional reaction. To this aim, we constructed parallel versions of selected jokes in such a way that all comprehension processes should remain constant apart from the processes of interest outlined above. This manipulation was realized by exchanging only the final word of the original jokes as in the following examples [compared to (1)]:

(2) –“Mummy, I just turned 14 years. May I please, finally, be allowed to wear a bra and make up.” –“No, you are not. And eat up your soup, my *girl*!”

(3) –“Mummy, I just turned 14 years. May I please, finally, be allowed to wear a bra and make up.” –“No, you are not. And eat up your soup, my *father*!”

In example (2), the interpretation of the whole discourse is straight-forward and coherent. Thus, no belief revision is necessary. In example (3), the initial interpretation gets violated. The final sentence is a grammatically and semantically correct sentence, but its final word is discourse incoherent, thus triggering revision processes. In contrast to the joke ending of example (1), no hidden interpretation (or at least no plausible one) can be activated and no alternative meaningful coherent representation of the text can be constructed. The joke endings share the discourse incoherence with (3) at the occurrence of the final word, but share the comprehensibility of a meaningful discourse with (2), once the belief revision has been successfully carried out. In a series of experiments, we investigated the neuro-cognitive processes being specific for GP jokes, using 48 GP jokes and their coherent and incoherent manipulations as stimuli. Experiment 1 focused on behavioral measures using a self-paced reading time paradigm. Here, we expected increasing reading times from coherent over incoherent to joke endings. In Experiments 2–4, ERPs were of main interest in order to localize the GP-specific sub-processes. Hypotheses were as follows: Joke endings and incoherent endings both represent the violation of the initially dominant semantic representation and should thus elicit an augmented N400 component. Successful belief-revision processes in GP joke comprehension—requiring enhanced inferential and working-memory related processes—should be reflected in the occurrence of LLAN components. Only joke endings should elicit an emotional response. Therefore, we expected emotion-related ERP components at subsequent, late stages of joke processing, namely following the violation and the revision processes.

Another potent indicator of both cognitive and emotional processes during the comprehension of jokes could be provided by pupillary responses, which we also measured in Experiment 2. First, changes of pupil diameter have been shown to be a sensitive measure of the cognitive load during a task: Higher cognitive load leads to larger pupil diameters (Kahneman and Beatty, 1966; van der Meer et al., 2010). Second, larger pupil diameters have also been reported in association with higher emotional involvement, related to the arousal (Bradley et al., 2008) or to the intensity (Partala and Surakka, 2003) of an emotional reaction. Both factors cognitive load and emotional processing have been shown to affect pupil diameters also in the processing of verbal stimuli, such as single word processing and recognition (Võ et al., 2008; Bayer et al., 2011). Since the successful comprehension of GP jokes is hypothesized to involve both increased cognitive processing effort and an emotional response, we expected larger pupil diameters after joke endings compared to coherent endings. Changes of pupil diameter to incoherent endings should be intermediate due to enhanced cognitive demands (violation detection) on the one hand but the absence of both revision processes and emotional response on the other hand.

## Experiment 1: reading times

The comprehension process of GP jokes is considered to contain two important stages: the detection of the violation of the semantic representation and the belief revision process. Both factors should lead to enhanced cognitive load which should be reflected in an increase of the reading times at the final word compared to coherent endings, as previously shown for English material (Coulson and Kutas, 1998). In the present experiment, we expected similar results for our German stimuli. Reading times for incoherent endings should be intermediate between coherent and joke endings since they also contain violations of the initial semantic representation that cannot be overcome by successful revision processes. However, given the equal contribution of all three stimulus categories, recipients might nevertheless attempt to search for potential hidden interpretations within their semantic system. Depending on how fast this search is interrupted, reading times might, alternatively, be even longer for incoherent compared to joke endings.

### Method

#### Participants

Twenty-four participants (16 females), ranging in age between 18 and 29 years (*M* = 22.48, *SD* = 2.93), were tested. All of them were native speakers of German and students at the University of Göttingen, coming from a wide range of disciplines. They were rewarded with 8 €/h for their participation.

### Material

A total number of 144 stimuli was constructed. Forty-eight jokes were selected from different sources according to the following criteria: (i) They had to exploit the GP mechanism. Additionally, they were selected to be (ii) ethically acceptable, (iii) subjectively amusing, (iv) translatable into German, unless they were originally German, without losing the amusement potential and without destroying the underlying GP structure, and (v) rewritable in such a way that the very final word of the last sentence could serve as the crucial punch-line element.

Based on these 48 jokes, two additional versions were constructed by exchanging only the final word of the text. In the Coherent condition, the final word of the joke was replaced by a word which was coherent according to the initial first interpretation of the text. In the Incoherent condition, the final word was replaced by a word which is incoherent according to the first interpretation and which does not offer a hidden interpretation of the set-up. Importantly, this final word violated neither the syntactic nor the semantic structure of the last sentence but it did not fit into the whole discourse of the text. This led to a total number of 144 stimuli with 48 text fragments identical in all three conditions but varying final words between conditions. Final words were matched between conditions according to word category, word frequency (Leipziger Worthäufigkeitsklasse; http://wortschatz.informatik.uni-leipzig.de/), and word length (number of letters). Descriptive statistics of the material is reported in Table 1. Stimulus material is provided as Supplementary Material.

**Table 1:** **Descriptive data of the stimulus features**.

**Variable**	***M***	***SD***
**(A) COHERENT**		
Number of letters	8.17	2.9
Word frequency	12.62	3.62
**(B) INCOHERENT**		
Number of letters	7.9	3.13
Word frequency	12.35	3.95
**(C) JOKE**		
Number of letters	7.81	2.94
Word frequency	11.88	3.9

In pre-experimental ratings, 68 participants (46 females) between 18 and 36 years (*M* = 23.19, *SD* = 3.38) evaluated on 5-step scales from 1 (do not agree at all/ trifft überhaupt nicht zu) to 5 (totally agree/ trifft völlig zu). Items were constructed according to three theoretically derived dimensions: humorous potential (Humor), predictability of the ending (Predictability), and comprehensibility of the whole text (Comprehensibility). For each dimension, three items were constructed in order to obtain: (i) a behavioral component, (ii) a cognitive appraisal, (iii) an emotional response.

(it.1) *The text was familiar, even though not necessarily literally. Der Text war mir zumindest sinngemäß bekannt*. (Familiarity).
(it.2) I did understand the text. *Ich habe den Text verstanden*. (Comprehension).
(it.3) The text made me laugh/smile. *Der Text hat mich zum Lachen/Schmunzeln gebracht*. (Humor Behavioral).
(it.4) The text amused me. *Der Text hat mich erheitert*. (Humor Emotional).
(it.5) The text is funny. *Der Text ist witzig*. (Humor Cognitive).
(it.6) The text tricked me into the wrong way. *Der Text hat mich in die Irre geleitet*. (Predictability Behavioral).
(it.7) The ending of the text did surprise me. *Das Ende des Textes hat mich überrascht*. (Predictability Emotional).
(it.8) The ending of the text is predictable. *Das Ende des Textes ist vorhersehbar*. (Predictability Cognitive).
(it.9) The text is understandable. *Der Text ist verständlich*. (Comprehensibility Behavioral).
(it.10) The text confused me. *Der Text hat mich verwirrt*. (Comprehensibility Emotional).
(it.12) The text is nonsense. *Der Text ist Unsinn*. (Comprehensibility Cognitive).

**Figure 1:**
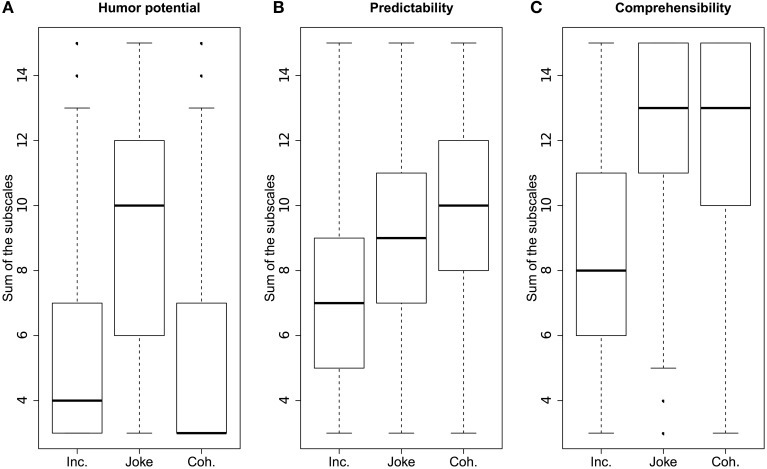
**Box plots of the three scales of the ratings, **(A)** Humor potential, **(B)** Predictability, and **(C)** Comprehensibility**. Every data point presents one observation for one participant and one stimulus. The thick line is the median, the box represents the 25% and 75% quantiles, and the whiskers are the minimum and maximum values, while points represent statistical outliers.

After reading the stimulus, participants indicated whether they knew the text, and then rated the nine items in randomized order. These three items per scale were summed together for the three total scale scores. The results of the ratings are depicted in Figure 1.

ANOVAs and Bonferroni-corrected *post-hoc t*-tests were carried out for the three scales. Only texts that were indicated as unfamiliar to the participants were included in the analysis. There was a significant effect of Condition on all three scales: Humor, *F*_(2, 141)_ = 135.31, *p* < 0.001, Predictability, *F*_(2, 141)_ = 77.48, *p* < 0.001, and Comprehensibility, *F*_(2, 141)_ = 115.45, *p* < 0.001. The Joke condition (*M* = 8.83, *SD* = 1.04) was rated as more humorous than both the Coherent (*M* = 5.39, *SD* = 1.42), *t*_(94)_ = 14.52, *p* < 0.001, and the Incoherent condition (*M* = 5.5, *SD* = 1.06), *t*_(94)_ = 13.29, *p* < 0.001, while there was no significant difference between Coherent and Incoherent. The Joke condition (*M* = 8.83, *SD* = 1.04) was rated less predictable than Coherent (*M* = 9.92, *SD* = 1.24), *t*_(94)_ = −4.66, *p* < 0.001, but more predictable than Incoherent (*M* = 7.07, *SD* = 1.11), *t*_(94)_ = 8.04, *p* < 0.001. The Incoherent condition (*M* = 8.33, *SD* = 1.68) was rated less comprehensible than the Joke condition (*M* = 12.25, *SD* = 1.12), *t*_(94)_ = −13.41, *p* < 0.001, and than the Coherent condition (*M* = 11.89, *SD* = 1.32), *t*_(94)_ = −11.51, *p* < 0.001, while there was no significant difference between Joke and Coherent conditions. Together, ratings confirmed the validity and the suitability of the stimulus material.

The 144 stimuli (48 Joke, 48 Coherent, 48 Incoherent) were used for Experiment 1. In addition, 144 filler items were constructed as similar as possible to the original stimuli in terms of the linguistic style, e.g., syntactic structure, topic, lexical level, dialogs, etc. Similar to the experimental stimuli, identical 48 text fragments were completed with three different endings: two different coherent endings and a discourse-incoherent ending. The filler items reduced the proportion of jokes in order to make the purpose of the study less obvious, they reduced the number of repetitions of the text fragments and, should, therefore, distract the participants from keeping all the text fragments in memory. Note that responses to fillers were not analyzed. The total of 288 texts was distributed to three different sets (every set containing 96 different text fragments). The order of the texts within a set was randomized for every participant and the six possible permutations of the block order were equally distributed over all the participants, resulting in 288 short texts of six conditions for each participant, guaranteeing that possible influences by the repetition of the text fragments were at least equally counterbalanced.

### Procedure

The experiment was carried out in a group lab on a computer with four participants per session. After they had indicated the demographic data, participants received instructions on the computer screen that they participated in an experiment on text comprehension. They were made familiar with the presentation of the stimuli and were told to carefully read the texts. They were explicitly told that some of the texts were hard to understand, and that some of them did not make sense at all. Also, they were explicitly instructed to continue with the next stimulus when they think that they understood the text or when they are sure that the text does not make sense.

The texts were presented on a computer screen with an adapted version of the Moving Windows Paradigm (Just et al., 1982), implemented by *Pygame*, a graphical interface for *Python*. First, the whole text was presented to the participant with the final sentence of the text being masked by blanks. The last sentence of the text appeared word by word after participants pressed the return key on a standard keyboard. Only the actual word appeared unmasked, and the words that had been read became masked again. Most importantly, the reading time for the final word (the crucial manipulation of the experiment) was measured as the time between the onset of the final word and the moment a participant pressed the return key on the keyboard in order to proceed with the next text.

After a pseudo-randomly chosen amount of trials (normal distribution with *M* = 10, *SD* = 4), participants were presented with a statement concerning the previously presented text and had to indicate whether the statement was true. The comprehension question was randomly chosen for correct “true” or correct “false” answers in order to prevent participants from clicking themselves through the task without proper processing of the stimuli.

### Results

Responses below 200 ms and above 3 standard deviations above participant's average were excluded from the analysis. Every participant's mean reading times of the final word per condition were calculated and log-transformed. Cohen's *d_z_* is reported as effect size as mean difference score per participant and condition divided by the standard deviations of these differences (Lakens, 2013). A One-Way ANOVA revealed a significant main effect of Condition, *F*_(2, 46)_ = 8.51, *p* < 0.001, η^2^_*G*_ = 0.27, with significantly shorter reading times for Coherent (*M* = 1018, *SD* = 329) as compared to both Joke (*M* = 1162, *SD* = 446), *t*_(23)_ = −3.97, *p* < 0.001, *d*_*z*_ = −0.81, and Incoherent (*M* = 1111, *SD* = 403), *t*_(23)_ = −2.62, *p* = 0.015, *d*_*z*_ = −0.53. The latter did not differ significantly, *t*_(23)_ = −1.49, *p* = 0.149, *d*_*z*_ = −0.30.

### Discussion

The hypothesis of longer reading times for joke endings compared to coherent endings was clearly supported by the data. Further, the reading times of the joke endings tended to be prolonged in comparison to incoherent endings, but this difference failed significance. Reading of incoherent endings took also significantly longer than of coherent endings. Together, these findings indicated that either the detection of the semantic incoherence itself is characterized by higher processing demands or the participants started the same attempt of finding an alternative interpretation as in the joke endings, possibly, triggered by the mere occurrence of jokes during the experiment.

## Experiment 2: evidence from ERPs and pupil diameters

Reading times, as measured in Experiment 1, reflect only the sum of several sub-processes, thus not allowing any specific assumptions regarding specific processing stages. ERPs provide the advantage of high temporal resolution in the range of milliseconds. Therefore, distinguishable ERP components can be related more precisely to the hypothesized underlying cognitive or emotional processing stages involved. Here, we recorded ERPs and changes of the pupil diameter in relation to the different endings of the stimulus material in order to investigate the hypothesized comprehension processes as outlined in the introduction.

### Method

#### Participants

Twenty-five students from different disciplines participated in this experiment. All of them were native speakers of German. From this sample, data of four participants had to be removed from analysis because they were familiar with too many of the jokes (*N* = 2) or because of excessive number of EEG artifacts (*N* = 2). The remaining 21 participants (14 females) were between 20 and 34 years old (*M* = 24.2, *SD* = 3.82) and had an LQ score between −90 and 100 (*M* = 60.4, *SD* = 57.34), according to the Edinburgh Handedness Inventory (Oldfield, 1971). All reported normal or corrected-to-normal vision and no neurological or neuropsychological disorders. Participants gave informed consent and received 20 € or course credits. None of them had participated in the rating experiment or in Experiment 1.

### Material

Exactly the same stimulus material was used as in Experiment 1.

### Procedure

Participants were seated in a dimly illuminated, sound-attenuated, and electrically shielded chamber, facing a monitor at a distance of 60 cm. They were made familiar with the presentation of the stimuli and were instructed to carefully read the texts. They were explicitly told that some of the texts are hard to understand and that some of them do not make sense at all. The texts were presented on the computer screen with an adapted version of an RSVP (rapid serial visual presentation) paradigm, implemented by *Pygame* in black on light-yellow background. Each trial consisted of the following sequence: The text fragment (without the final sentence) was presented at the center of the screen. After a button press, the final sentences began with a fixation cross of 500 ms duration. They were presented word-by-word, with 250 ms duration per word and 500 ms SOA. After the critical final word disappeared, another fixation cross was presented for 5000 ms, which was followed by a comprehension task. A statement concerning the preceding text was presented and participants were asked to indicate whether the statement is correct or not by pressing one of two buttons. The questions were pseudo-randomly chosen to be either correctly accepted or declined. Afterwards, participants had to indicate on a questionnaire which of the jokes they were sure that they had been familiar with prior the experiment.

### Psychophysiological recordings, processing, and analysis

#### ERPs

ERPs were recorded from 68 active Ag/AgCl electrodes located according to the extended 10–20 system (Picton et al., 2000). Sixty-four electrodes were placed in an electrode cap. External electrodes were used for the vertical and horizontal electrooculogram (left eye) and left and right mastoid. EEG signals were amplified by a Biosemi ActiveTwo AD-box, referenced to the common mode sense (CMS; active electrode) and grounded to the driven right leg (DRL; passive electrode). All electrodes were recorded with a passband of 0.16–100 Hz; sampling rate was 512 Hz. Offline, the continuous EEG record was converted to average reference^3^, corrected for blinks using Surrogate Multiple Source Eye Correction (MSEC; Ille et al., 2002) as implemented in BESA (Brain Electric Source Analysis, MEGIS Software GmbH) and filtered with a 30 Hz low-pass filter. Continuous EEG data was segmented into epochs of 1200 ms, starting 200 ms before the onset of the critical (final) word. All ERPs were referred to a 200 ms pre-stimulus baseline. Epochs containing artifacts were automatically discarded when any amplitude exceeded −100 or +100 μV or when any voltage step exceeded 50 μV per sampling point in any of the electrodes. Data of two participants were dropped from analyses (less than 50% of the trials remained). For all remaining participants, between 61% and 100% of the trials (*M* = 89.94, *SD* = 10.82) entered the analysis, with the additional criteria of correct responses to comprehension questions and indicated unfamiliarity of jokes. In total, 20–37 (*M* = 29.1, *SD* = 5.8) trials per participant, and experimental condition were averaged. All ERPs were referred to a 200 ms pre-stimulus baseline.

**Figure 2:**
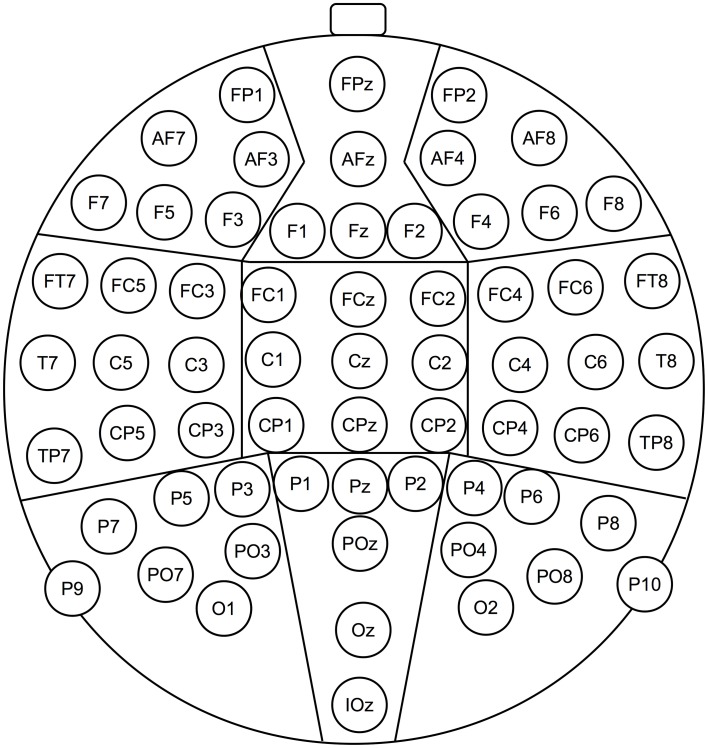
**Schematic depiction of the 64 electrodes and their approximate locations, as used in Experiments 2–4**. The lines demarcate the nine electrode clusters, dividing the whole set of electrodes into three levels each of the factors Caudality and Laterality involved in ERP analyses.

Based on the literature and on visual inspection of the data, mean ERP amplitudes were calculated in the three following time windows after stimulus onset: 250–500 ms (N400), 500–700 ms (LLAN), and 700–1000 ms (late positivities). The 64 electrodes were grouped into nine clusters (see Figure 2). Data were then submitted to repeated measures overall ANOVAs including the factors Condition (Coherent, Incoherent, Joke), Laterality (Left, Midline, Right) and Caudality (Anterior, Central, Posterior). Significant interaction effects were followed up by step-down ANOVAs. Finally, repeated measures ANOVAs were calculated for each electrode cluster separately. In all analyses, Greenhouse-Geisser correction was applied to adjust the degrees of freedom of the F-ratios if the sphericity assumption was violated according to the Mauchly test. Please note that all within-subject repeated ANOVA measures will be reported with uncorrected degrees of freedom but Greenhouse-Geisser corrected *p*-values if indicated by the Mauchly test. In all cases, for multiple *post-hoc* comparisons alpha levels were Bonferroni-corrected.

#### Pupil diameters

Pupil diameters were continuously tracked with a Desktop Mount Eye-tracking System, EYELINK 2000 by SR Research. The method was elliptic tracking of the dominant eye at a 50% illumination rate and a 1000 Hz sampling rate. The head position was stabilized with a chin and a forehead rest. Each block of the experiment was started with a 9-point-calibration and validation phase of the eye tracking. Offline, continuous data were segmented into epochs of 3200 ms, starting 200 ms before the onset of the final word; segments were referred to the 200 ms pre-stimulus interval. Incorrectly answered trials and trials with jokes that were familiar to the participants before the experiment were removed. Trials with blinks were removed; the missing data was interpolated with the preceding and following 50 samples. ANOVAs with Condition as a within-factor (three levels) were conducted on averaged data in consecutive 50-ms segments in order to detect the onsets of significant differences. Bonferroni-corrected *post-hoc t*-tests for paired samples were further applied in case of significant main effects. The data from participants excluded from ERP analysis did not enter these analyses.

### Results

#### Behavioral data

The test scores are presented in Table 2. ANOVA on Comprehension scores showed a significant effect of Condition, *F*_(2, 40)_ = 29.58, *p* < 0.001, η^2^_*p*_ = 0.59, with significantly more correctly answered trials in the Coherent than in both the Joke, *t*_(20)_ = 6.59, *p* < 0.001, *d*_*z*_ = 1.52, and Incoherent condition, *t*_(20)_ = 8.13, *p* < 0.001, *d*_*z*_ = 1.8. There was no difference in comprehension accuracy between the Joke and the Incoherent condition, *t*_(20)_ = −0.82, *p* = 0.430, *d*_*z*_ = −0.17.

**Table 2:** **Descriptive data of the Comprehension score in Experiment 2 (*N* = 21)**.

**Variable**	***M***	***SD***
Comprehension total	85.52	3.33
Comprehension coherent	88.59	3.99
Comprehension incoherent	77.78	5.94
Comprehension joke	76.19	8.03
Number of familiar jokes	7.9	6.38

#### Electrophysiological data

**Figure 3:**
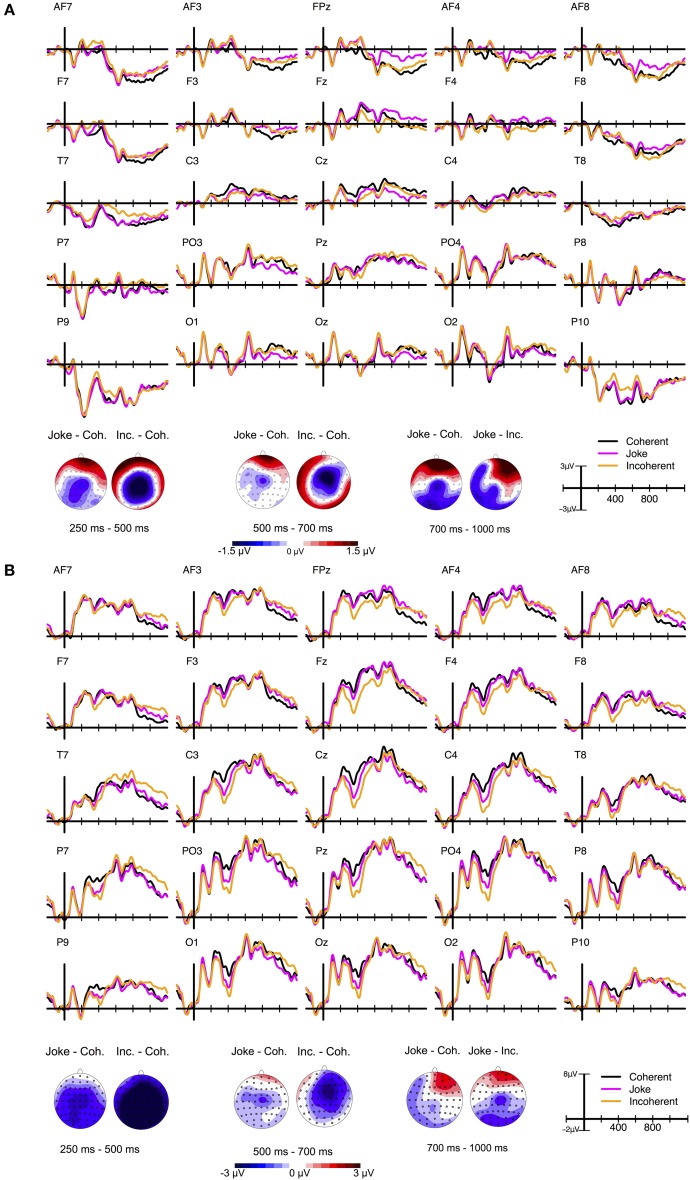
**ERP grand average waves for selected electrodes as a function of time relative to the stimulus onset and Condition in Experiment 2 and corresponding ERP difference maps for three selected time windows**. **(A)** Depicts data recalculated to average reference (AR) data and **(B)** data recalculated to mastoid reference (MR). As becomes obvious from a comparison of the ERP difference maps depicted in **(A)** and **(B)**, different types of references do not alter the topography of ERPs across the scalp but only affect the zero line (Michel et al., 2004). The choice of reference thus influences the magnitude characteristics of ERPs at certain electrode sites (cf. Lehmann, 1977; Rellecke et al., 2013), as can be seen in the ERP grand average waveforms. By definition, the voltage of any potential on the scalp is set to zero at the location of the reference electrode(s). In case of the N400 component, the application of MR to the ERPs shifts the zero voltage line toward positivities, resulting in broader centro-parietal distributions of the relative negativities, resembling the N400 component as depicted in previous reports (see Koenig and Gianotti, 2009; for details regarding the choice of appropriate references).

ERP grand averages and maps of ERP differences between conditions are depicted in Figure 3, recalculated to average reference (panel A) and mastoid reference (panel B). Please note that all statistics reported in the present paper are based on average-referenced ERPs.

#### N400 (250–500 ms)

The repeated measures omnibus-ANOVA including the factors Condition (Joke, Coherent, Incoherent), Laterality (Left, Midline, Right), and Caudality (Anterior, Central, Posterior) revealed a significant main effect of Condition, *F*_(2, 40)_ = 3.41, *p* = 0.043, ε = 0.802, η^2^_*p*_ = 0.194, as well as significant interactions between Condition and Laterality, *F*_(4, 80)_ = 6.87, *p* < 0.001, ε = 0.705, η^2^_*p*_ = 0.487, and between Condition and Caudality, *F*_(4, 80)_ = 4.03, *p* = 0.018, ε = 0.588, η^2^_*p*_ = 0.362. There was a tendency for an interaction effect between Laterality, Caudality, and Condition, *F*_(8, 160)_ = 2.33, *p* = 0.053, ε = 0.574, η^2^_*p*_ = 0.723.

Follow-up repeated measures ANOVAs (Bonferroni corrected) at each of the three levels of Caudality and Laterality separately revealed: a tendency for a main effect of Condition in Anterior, *F*_(2, 40)_ = 4.71, *p* = 0.014, ε = 0.884, η^2^_*p*_ = 0.272, as well as in Left, *F*_(2, 40)_ = 4.63, *p* = 0.015, ε = 0.827, η^2^_*p*_ = 0.334. Significant main effects of Condition were obtained in Central, *F*_(2, 40)_ = 8.94, *p* < 0.001, ε = 0.790, η^2^_*p*_ = 0.371, and in Midline, *F*_(2, 40)_ = 11.49, *p* < 0.001, ε = 0.787, η^2^_*p*_ = 0.464. Effects of Condition were neither significant in Posterior, *F*_(2, 40)_ = 2.01, *p* = 0.147, ε = 0.869, η^2^_*p*_ = 0.216, nor in Right, *F*_(2, 40)_ = 1.61, *p* = 0.212, ε = 0.958, η^2^_*p*_ = 0.122.

The effect was strongest at electrodes around Cz, *F*_(2, 40)_ = 15.57, *p* < 0.001, ε = 0.744, η^2^_*p*_ = 0.529. A pairwise *post-hoc* comparison (Bonferroni corrected) for this central-midline region revealed significant differences between all three levels: Incoherent vs. Coherent, *t*_(20)_ = −4.45, *p* < 0.001, *d*_*z*_ = −0.97, Joke vs. Coherent, *t*_(20)_ = −4.09, *p* < 0.001, *d*_*z*_ = −0.89, Incoherent vs. Joke, *t*_(20)_ = −2.86, *p* = 0.009, *d*_*z*_ = −0.62.

#### LLAN (500–700 ms)

In the following interval from 500 to 700 ms, the repeated measures omnibus-ANOVA revealed a significant interaction effect between Laterality and Condition, *F*_(4, 80)_ = 11.35, *p* < 0.001, ε = 0.705, η^2^_*p*_ = 0.487, and an interaction effect between Laterality, Caudality, and Condition, *F*_(8, 160)_ = 3.11, *p* = 0.016, ε = 0.549, η^2^_*p*_ = 0.485. No significant effect was found for Condition, *F*_(2, 40)_ = 2.33, *p* = 0.12, ε = 0.839, η^2^_*p*_ = 0.204. There was a tendency for a Caudality by Condition interaction, *F*_(4, 80)_ = 3.01, *p* = 0.053, ε = 0.565, η^2^_*p*_ = 0.395.

Follow-up repeated measures ANOVAs at each of the three levels of Laterality separately revealed: In Left, a significant main effect of Condition, *F*_(2, 40)_ = 19.71, *p* < 0.001, ε = 0.920, η^2^_*p*_ = 0.603, but no Caudality by Condition interaction, *F*_(4,80)_ < 1. In Midline, both a significant main effect of Condition, *F*_(2, 40)_ = 8.36, *p* = 0.002, ε = 0.775, η^2^_*p*_ = 0.407, and a significant Caudality by Condition interaction, *F*_(4, 80)_ = 4.15, *p* = 0.016, ε = 0.598, η^2^_*p*_ = 0.454. In Right, a significant main effect of Condition, *F*_(2, 40)_ = 5.53, *p* = 0.007, ε = 0.965, η^2^_*p*_ = 0.377, and a tendency for a Caudality by Condition interaction, *F*_(4, 80)_ = 3.19, *p* = 0.051, ε = 0.562, η^2^_*p*_ = 0.256.

ANOVAs for all three midline regions separately, showed that the effect of Condition was again strongest for the central region around Cz electrode, *F*_(2, 40)_ = 8.04, *p* = 0.001, ε = 0.798, η^2^_*p*_ = 0.449, but also significant in the anterior-midline region, *F*_(2, 40)_ = 7.49, *p* = 0.001, ε = 0.943, η^2^_*p*_ = 0.382, but not in the posterior-midline region. A pairwise *post-hoc* comparison (Bonferroni corrected) for this central-midline region revealed significant differences between Incoherent and Coherent, *t*_(20)_ = −3.6, *p* = 0.002, *d*_*z*_ = −0.79, Joke and Coherent, *t*_(20)_ = −2.81, *p* = 0.011, *d*_*z*_ = −0.61, but not between Incoherent and Joke, *t*_(20)_ = −1.8, *p* = 0.086, *d*_*z*_ = −0.39.

#### Late positivities (700–1000 ms)

In the following interval from 700 to 1000 ms, the repeated measures omnibus-ANOVA revealed a significant interaction effect between Laterality and Condition, *F*_(4, 80)_ = 5.66, *p* < 0.001, ε = 0.809, η^2^_*p*_ = 0.570, between Caudality and Condition, *F*_(4, 80)_ = 5.40, *p* = 0.005, ε = 0.591, η^2^_*p*_ = 0.558, and an interaction effect between Laterality, Caudality, and Condition, *F*_(8,160)_ = 2.94, *p* = 0.019, ε = 0.573, η^2^_*p*_ = 0.693. No significant effect was found for Condition, *F*_(2,40)_ < 1.

Follow-up repeated measures ANOVAs at each of the three levels of Laterality separately revealed: in Left, both a significant main effect of Condition, *F*_(2, 40)_ = 10.27, *p* < 0.001, ε = 0.881, η^2^_*p*_ = 0.494, and a tendency for a Caudality by Condition interaction, *F*_(4, 80)_ = 3.82, *p* = 0.025, ε = 0.557, η^2^_*p*_ = 0.271. In Midline, Condition had no main effect, *F*_(2,40)_ < 1, but significantly interacted with Caudality, *F*_(4, 80)_ = 5.35, *p* = 0.004, ε = 0.617, η^2^_*p*_ = 0.619. In Right, there were both a significant main effect of Condition, *F*_(2, 40)_ = 7.17, *p* = 0.003, ε = 0.899, η^2^_*p*_ = 0.404, and a significant Condition by Caudality interaction, *F*_(4, 80)_ = 4.62, *p* = 0.011, ε = 0.588, η^2^_*p*_ = 0.436.

ANOVAs for all three nine regions separately revealed a significant main effect of Condition for all three anterior regions and for the central-left region. However, this effect was strongest for the anterior-right region, *F*_(2, 40)_ = 8.12, *p* = 0.001, ε = 0.832, η^2^_*p*_ = 0.568, due to significant differences between Joke and Coherent, *t*_(20)_ = 4.27, *p* < 0.001, *d*_*z*_ = 0.93, and between Joke and Incoherent, *t*_(20)_ = 3.58, *p* = 0.002, *d*_*z*_ = 0.78, but not between Incoherent and Coherent, *t*_(20)_ = −0.34, *p* = 0.732, *d*_*z*_ = −0.07.

#### Pupil diameter data

**Figure 4:**
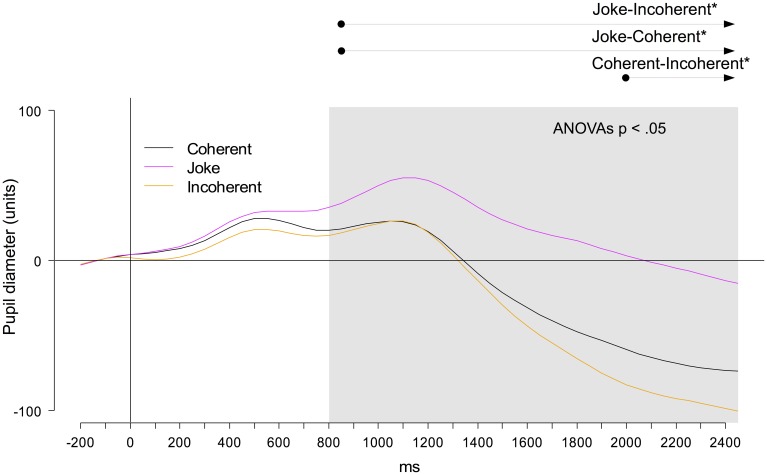
**Changes in pupil diameters (arbitrary unit) as a function of time relative to the stimulus onset and Condition**. The gray box indicates the time window with significant main effects of Condition, as revealed by running ANOVAs in consecutive 50 ms-steps. The arrows indicate the time windows with Bonferroni-corrected significant *post-hoc* comparisons.

An ANOVA of mean pupil diameters in consecutive segments of 50 ms, including the factor Condition, revealed significant differences between 800 and 3000 ms, *F*s_(2,40)_ = 4.30–30.15, *p*s = 0.049 to <0.001, η^2^_*p*_s = 0.160–0.600. Bonferroni-corrected *t*-tests indicated significantly larger pupil diameters after joke endings compared to both incoherent and coherent endings, starting at 850 ms post onset and persisting until the end of segmentation, *t*s_(20)_ = 2.57–6.27, *p*s = 0.016 to <0.001, *d*_*z*_s = 0.52–1.36 (see Figure 4). Further, larger pupil diameters were elicited by coherent compared to incoherent endings, between 2000 ms and the end of segmentation, *t*s_(20)_ =2.55–3.03, *p*s = 0.016–0.006, *d*_*z*_s = 0.52−0.65. We also calculated a Pearson correlation of the pupil diameter data of joke endings (aggregated over all participants) with the three scales of the pre-rating for each joke. Pupil diameter data only significantly correlated with the rating for the humorous potential, *r*_(46)_ = 0.43, *p* = 0.003, but not with the other two scales: Predictability, *r*_(46)_ = 0.174, *p* = 0.251, Comprehensibility, *r*_(46)_ = 0.005, *p* = 0.972.

### Discussion

As hypothesized and in line with previous findings (Derks et al., 1997; Coulson and Kutas, 2001), joke endings elicited more negative ERP amplitudes at central electrode sites compared to the coherent endings between 250 and 500 ms after the onset of the final word. As can be seen in Figure 3A, this increased negativity over the vertex was accompanied by a frontal positivity, reflecting the polarity reversal of centro-parietal negativity due to the average reference applied here. Compared to incoherent endings, this N400 effect was reduced in the Joke condition. The N400 component is a reliable measurement for the degree of expectation violation and, even more important, for the degree of semantic integration difficulties. Therefore, the N400 effect here paralleled the predictability ratings of Experiment 1. The finding of reduced N400 effects to joke as compared to incoherent endings therefore suggests a less-salient violation and, probably, the activation of a coherent alternative hidden joke interpretation. This activation might initiate a spreading activation toward new relevant semantic content for rapidly integrating the joke endings into the context. In contrast, for completely incoherent endings, such an activation and integration of a possible alternative interpretation might not occur.

In the time window of the hypothesized LLAN, i.e., 500–700 ms after the onset of the final word, no evidence for a joke-related LLAN component was found. The LLAN component had been hypothesized to reflect increased working memory load necessary for the re-establishment of a coherent discourse or successful “frame-shifting” (Coulson and Kutas, 2001; Coulson and Lovett, 2004). Instead, we mainly found a sustained N400 effect, consisting in an enhanced negativity over the vertex, for incoherent and joke endings during this time interval. In terms of amplitude differences, this sustained negativity was weaker for joke compared to incoherent endings. A possible reason for the lack of LLAN effects in the present study might be the mere presence of incoherent endings. Incoherent endings might have provided such a strong contrast to the two other conditions and, in particular, to the joke endings that the assumed processes of frame shifting following incoherence detection might have been suppressed. In addition, one might argue that the repetition of the text-fragments triggered the search for hidden joke interpretations also for incoherent endings. Accordingly, the LLAN might have been drastically reduced compared to previous studies (Coulson and Kutas, 2001; Coulson and Lovett, 2004). We directly tested for this possibility in Experiment 3.

In the time window of Late Positivity components (700–1000 ms), joke endings elicited a frontal positivity compared to both coherent and incoherent endings. Importantly, the anterior locus of this ERP effect clearly differed from the typical posterior scalp distribution of a P600. Our finding of such late positivity with an anterior rather than posterior maximum parallels a previous report by Coulson and Lovett (2004) for right-handed women during joke comprehension. The authors related this finding to less hemispheric lateralization in women than in men. Even though participants' handedness and sex were not equally balanced in the present study, the present sample consisted for a big part of right-handed women. Thus, it cannot be excluded that a more heterogeneous sample will lead to a more posterior positivity.

Alternatively, it seems plausible that this frontal late positivity reflected emotional processes during joke comprehension, corresponding to recent findings by Du et al. (2012). Accordingly, the frontal late positivity found here might be related to an “elaboration” stage of the joke comprehension, including emotional sub-processes toward the humorous stimuli, presumably generated in the vmPFC, bilateral amygdalae, and bilateral parahippocampal gyri (see Chan et al., 2012). Such interpretation is supported by our data from pupillometric recordings that parallel ERP findings: In both parameters, joke endings differed from both coherent and incoherent endings with larger pupil diameters and larger ERP amplitudes. Interestingly, pupil diameters for incoherent endings did initially not significantly differ from those to coherent endings. Starting later in time (around 2000 ms), however, pupil diameters were diminished after incoherent compared to coherent endings. This finding suggested either a lack of sustained cognitive processing effort or of emotional responses or even both in the Incoherent condition.

Together, two unexpected findings in our ERP data needed further clarification—the lack of LLAN effects to joke endings and of late posterior positivities expected to occur after incoherent endings. Both insignificances might be due to contextual effects caused by the experimental setting, as we have discussed above. In order to control for such potential context effects on the ERP effects obtained here, we conducted two additional experiments in which either the joke or the Incoherent condition were contrasted to coherent processing separately. Therefore, we omitted the Incoherent condition in Experiment 3 and the Joke condition in Experiment 4.

## Experiment 3: ERP comparison between joke and coherent processing

The results regarding joke endings from Experiment 2 needed further exploration for several reasons. Firstly, the previously reported enhanced LLAN to joke endings compared to coherent endings could not be replicated. Secondly, it could not be excluded that the mere presence of the incoherent endings affected the way participants processed the joke endings in a severe manner. In order to address these two points, Experiment 3 was carried out as a closer replication of a study by Coulson and Kutas (2001). Here, participants only received joke and coherent endings of the same text fragments. In addition, only right-handed participants were tested, and the distribution of male and female participants was equally balanced. We expected the following ERP effects: In comparison to coherent endings, joke endings should elicit enhanced amplitudes of the N400 and late positivities. If a mere presence of incoherent endings was responsible for the lack of a previously reported LLAN component, the LLAN should be elicited by joke endings under the current experimental conditions where incoherent endings were eliminated.

### Method

#### Participants

Twenty-four students (12 females, 12 males) from different disciplines, ranging in age between 19 and 28 years (*M* = 23.42, *SD* = 2.36) participated in this experiment. All of them were native speakers of German. They were all right-handed (Oldfield, 1971). All participants reported normal or corrected-to-normal vision and no neurological or neuropsychological disorders. Participants gave their informed consent and received 15 € or course credits. None of them had participated in one of the previously described experiments.

### Material and procedure

Exactly the same stimulus material was used as in Experiment 2, but the Incoherent stimuli and the Incoherent filler items were excluded, resulting in three blocks with 64 trials each. Stimulus presentation followed the procedure of Experiment 2.

#### EEG recording and analysis

Recording and pre-processing of the ERP data, including elimination of trials, artifact rejection, and definition of ERP components (time windows and ROI electrodes), followed the same procedures as that in Experiment 2. For all participants, between 59% and 89% of the trials (*M* = 70.70, *SD* = 14.18) remained for the analysis. ANOVAs on mean ERP amplitudes included the factors Laterality (Left, Midline, Right), Caudality (Anterior, Central, Posterior), and Condition (Joke, Coherent).

### Results

#### Behavioral data

Test scores are presented in Table 3. A paired samples *t*-test on comprehension scores showed a significant effect of Condition, *t*_(23)_ = −6.11, *p* < 0.001, *d*_*z*_ = 1.13, indicating worse comprehension of joke in comparison to coherent endings.

**Table 3:** **Descriptive data of the Comprehension score in Experiment 3 (*N* = 24)**.

**Variable**	***M***	***SD***
Comprehension total	88.35	4.55
Comprehension coherent	89.76	5.79
Comprehension joke	80.64	8.37
Number of familiar jokes	6.71	5.93

#### Electrophysiological data

**Figure 5:**
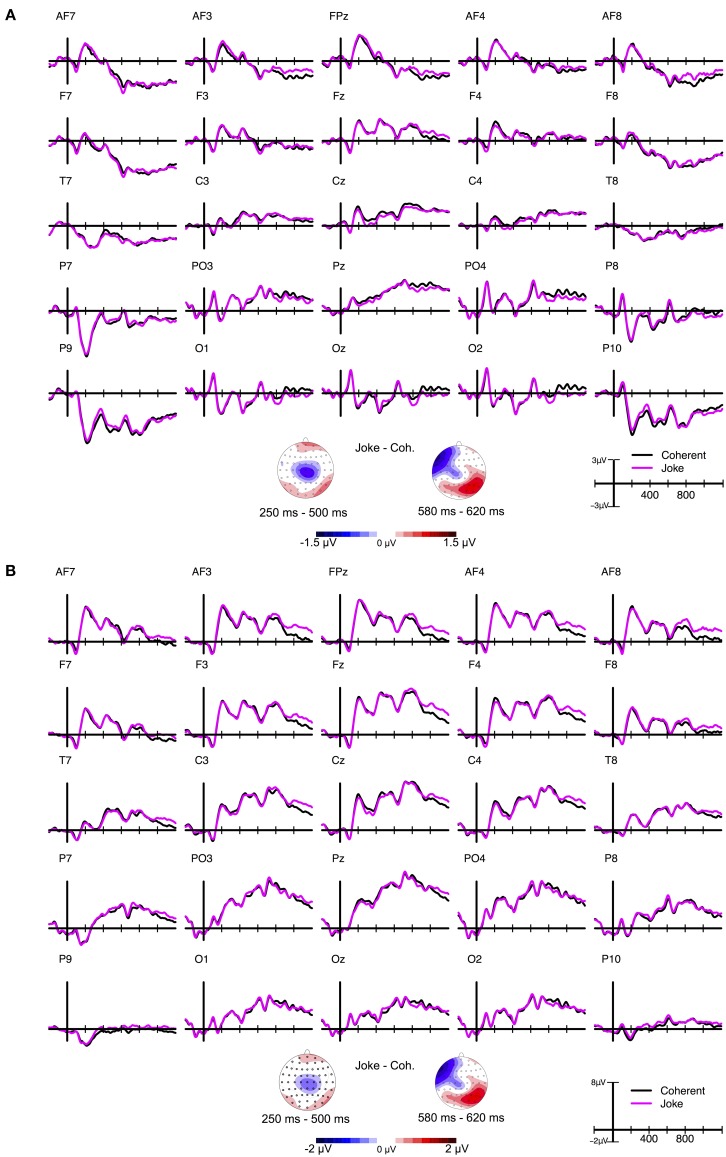
**ERP grand average waves for selected electrodes as a function of time relative to the stimulus onset and Condition in Experiment 3 and corresponding ERP difference maps for three selected time windows**. **(A)** depicts data recalculated to average reference (AR) data and **(B)** data recalculated to mastoid reference (MR).

ERP grand averages and maps of ERP differences between conditions are depicted in Figure 5, recalculated to average reference (panel A) and mastoid reference (panel B).

#### N400 (250–500 ms)

The repeated measures omnibus-ANOVA neither revealed a significant main effect of Condition, *F*_(1, 23)_ = 2.02, *p* = 0.168, η^2^_*p*_ = 0.081, nor any two- or three-way interactions, *F*s ≤ 1.68, *p* ≥ 0.206.

However, based on the a-priori hypothesis about an N400 effect and its indication in Experiment 2, we analyzed the central-midline electrode cluster for potential effects of Condition. Indeed, this analysis revealed a significant main effect of Condition, *t*_(23)_ = −2.93, *p* = 0.003, *d*_*z*_ = − 0.59, consisting in enhanced negative amplitudes to joke compared with coherent endings (see Figure 5).

#### LLAN (500–700 ms)

The repeated measures omnibus-ANOVA revealed neither a significant main effect of Condition, *F*_(1,23)_ < 1, nor any two- or three-way interactions, *F*s < 1.

Visual inspection of the data, however, indicated enhanced negative ERP amplitudes for joke endings that were restricted to a few left-anterior electrodes between 580 and 620 ms after the stimulus onset (see Figure 5). Since we were a-priori interested in any possible LLAN effect, we calculated a repeated measures ANOVA, including Condition and Electrode, on mean ERP amplitudes at these selected anterior electrodes (F7, F5, FT7, and AF7). The ANOVA revealed a significant main effect of Condition, *F*_(1, 23)_ = 4.89, *p* = 0.037, ε = 1, η^2^_*p*_ = 0.176, but no significant Condition by Electrode interaction, *F*_(3,69)_ < 1.

#### Late positivities (700–1000 ms)

The repeated measures omnibus-ANOVA, including the factors Condition (Joke, Coherent), Laterality (Left, Midline, Right), and Caudality (Anterior, Central, Posterior), neither revealed a significant main effect for Condition, *F*_(1, 23)_ = 1.39, *p* = 0.249, η^2^_*p*_ = 0.057, nor any two-way interactions, *F*s_(2,46)_ < 1, nor the three-way interaction between Laterality, Caudality, and Condition, *F*_(4, 92)_ = 1.9, *p* = 0.116, ε = 0.563, η^2^_*p*_ = 0.211.

### Discussion

First of all, it is striking that the effects obtained in Experiment 3 were—in general—weaker than the effects in Experiment 2, even though the same stimuli and the same procedure had been employed. There were two differences between these two experiments: the absence of the Incoherent condition, including incoherent fillers, and dissimilarities between both samples in terms of participants' variables (handedness and sex ratio). However, neither visual inspection nor statistical tests of the data revealed any sex-related differences. The first modification, that is elimination of incoherent endings, reduced the duration of the experimental session and might have also made the design of the experiment more obvious. Furthermore, each participant read every stimulus twice but with different endings. Although we aimed to reduce the importance of the repetition effect by a latin-squared design, it is difficult to estimate how the reduction by one level in the experimental design might have interacted with these possible repetition effects.

The expected N400 effect was not reliably found in Experiment 3. Only an analysis, based on the a-priori focus of the relevant ROI, revealed an impact of the experimental manipulation on the N400. Overall, the effect was also weaker than in Experiment 3. Since there were no incoherent endings, participants might have found it easier to semantically integrate the joke ending which led to less comprehension difficulties. Visual inspection of the data revealed similar activation patterns in the following time windows compared to Experiment 2 and to the literature (left anterior negativity and late frontal positivity). Statistical analysis, however, showed no convincing evidence for a negativity at left-anterior electrode sites between 500 and 700 ms (LLAN effect), nor for a frontal positivity between 700 and 1000 ms in the Joke condition. Restricted to a small time window between 580 and 620 ms after stimulus onset, joke endings elicited a left-frontal negativity accompanied by a contralateral posterior positivity, and thus showing strong similarities to LLAN effects in previous reports. Nevertheless, the reliability of this finding is highly questionable due to possible inflation of the type 1 error by the *post-hoc* selection of the time window and the electrodes.

## Experiment 4: ERP comparison between incoherent and coherent processing

In Experiment 2, incoherent endings as compared to coherent endings elicited larger and prolonged N400 effects as hypothesized but failed to evoke any late positivity. The data, however, showed a tendency for a right-anterior late positivity between 700 and 1000 ms, which was almost significant. As discussed above, the processing differences between the Incoherent and Coherent condition might have been affected by the mere presence of joke endings. While the N400 effect occurred as predicted, the late positivity showed a scalp topography that did not resemble the typical P600 component. Two explanations for the elicited frontal positivity in Experiment 2 seemed reasonable. On the one hand, it could be the case that the context of the humorous stimuli in Experiment 2 has severely altered the processing of incoherent endings. It might have caused participants to engage in the vain effort of searching a coherent interpretation for a nonsensical discourse. Such unsuccessful attempts are plausible if participants could not be completely confident about the incoherent endings being indeed incoherent or representing joke endings that were very hard to grasp. On the other hand, the anterior shift of the late positivity might have reflected increased frontal activity caused by the confusion after an incoherent discourse. Experiment 4 aimed to shed light on these questions by omitting all joke endings as well as both joke and incoherent filler endings.

### Method

#### Participants

Twenty-four students (12 females, 12 males) of different disciplines between 19 and 29 years (*M* = 22.79, *SD* = 2.43) participated in this experiment. All of them were native speakers of German and right-handed (according to Oldfield, 1971). All reported normal or corrected-to-normal vision and no neurological or neuropsychological disorders. Participants gave their informed consent and received 15 € or course credits. None of them had participated in one of the previously described experiments.

### Material and procedure

Exactly the same stimulus material was used as in Experiment 2, but the Joke stimuli and the Incoherent filler items were excluded (three blocks with 64 trials per block). Experimental procedure followed Experiment 2.

#### EEG recording and analysis

Recording and pre-processing of the ERP data followed Experiment 2. Between 58% and 100% of the trials (*M* = 94.36, *SD* = 8.7) remained after artifact rejection. ERP components of interest (N400, sustained N400, and late positivities), respective time windows (250–500 ms, 500–700 ms, and 700–1000 ms) as well as analyses were identical to Experiment 2, apart from only two levels of the factor Condition.

### Results

#### Behavioral data

The test scores are presented in Table 4. A paired samples *t*-test with Condition as a within factor and Comprehension score as the dependent variable showed a significant effect of Condition, *t*_(23)_ = −7.5, *p* < 0.001, *d*_*z*_ = −1.13, reflecting significantly lower comprehension score of Incoherent compared to Coherent.

**Table 4:** **Descriptive data of the Comprehension score in Experiment 4 (*N* = 24)**.

**Variable**	***M***	***SD***
Comprehension total	87.92	4.74
Comprehension coherent	89.65	4.91
Comprehension incoherent	78.15	8.91

#### Electrophysiological data

**Figure 6:**
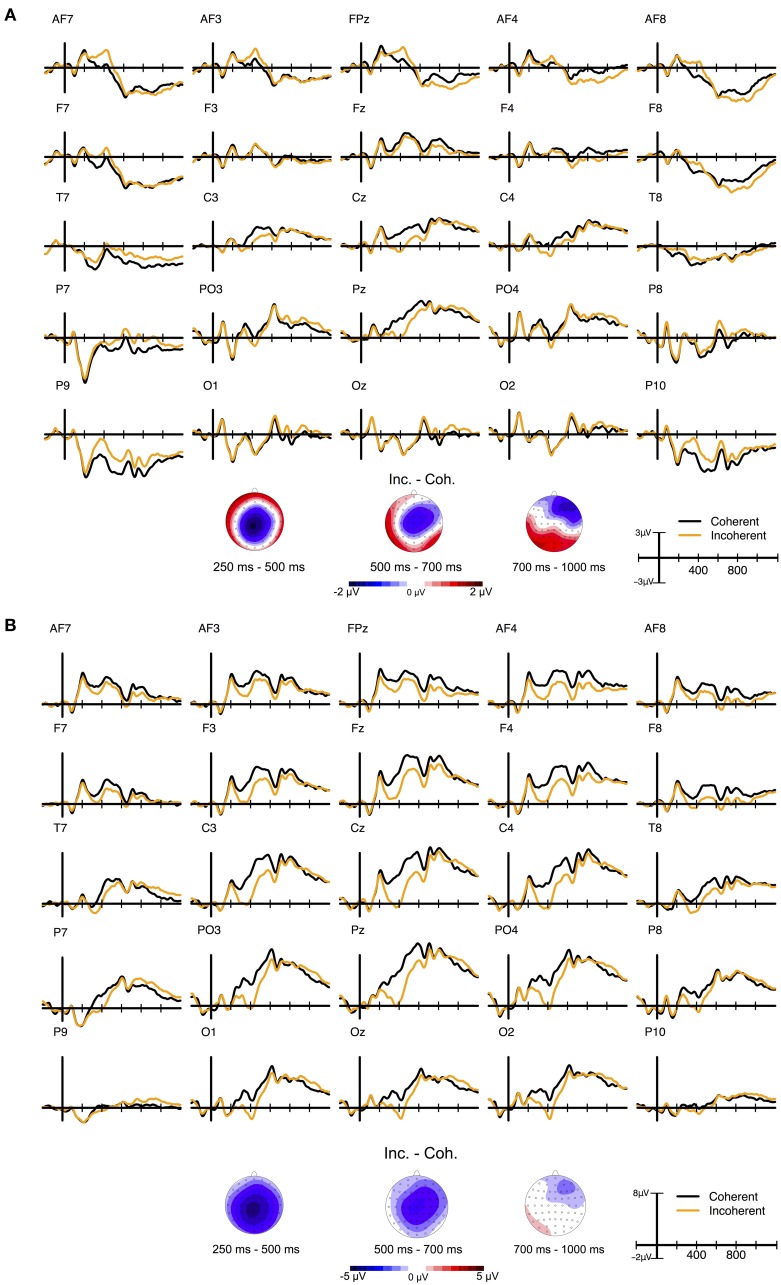
**ERP grand average waves for selected electrodes as a function of time relative to the stimulus onset and Condition in Experiment 4 and corresponding ERP difference maps for three selected time windows**. **(A)** depicts data recalculated to average reference (AR) data and **(B)** data recalculated to mastoid reference (MR).

ERP grand averages and maps of ERP differences between conditions are depicted in Figure 6, recalculated to average reference (panel A) and mastoid reference (panel B).

#### N400 (250–500 ms)

The repeated measures omnibus-ANOVA revealed a significant main effect of Condition, *F*_(1, 23)_ = 39.46, *p* < 0.001, η^2^_*p*_ = 0.631, as well as significant interactions between Laterality and Condition, *F*_(2, 46)_ = 20.43, *p* < 0.001, ε = 0.921, η^2^_*p*_ = 0.676, between Caudality and Condition, *F*_(2, 46)_ = 8.08, *p* = 0.005, ε = 0.608, η^2^_*p*_ = 0.696, and a significant three-way interaction between Laterality, Caudality, and Condition, *F*_(4, 92)_ = 4.41, *p* = 0.002, ε = 0.756, η^2^_*p*_ = 0.646.

Follow-up repeated measures ANOVAs for the three levels of Laterality separately revealed: in Left, both a significant main effect of Condition, *F*_(1, 23)_ = 15.75, *p* < 0.001, η^2^_*p*_ = 0.334, and a tendency for a Caudality by Condition interaction, *F*_(2, 46)_ = 5.25, *p* = 0.020, ε = 0.676, η^2^_*p*_ = 0.440. In Midline, there were both a significant main effect of Condition, *F*_(1, 23)_ = 43.74, *p* < 0.001, η^2^_*p*_ = 0.650, and a significant Caudality by Condition interaction, *F*_(2,46)_, = 11.22, *p* < 0.001, ε = 0.674, η^2^_*p*_ = 0.722. In Right, there was a tendency for a main effect, *F*_(1, 23)_ = 3.66, *p* = 0.068, η^2^_*p*_ = 0.137, and a tendency for a Caudality by Condition interaction, *F*_(2, 46)_ = 5.03, *p* = 0.027, ε = 0.605, η^2^_*p*_ = 0.501. The ERP difference between Incoherent and Coherent was strongest for the central-midline region around Cz electrode, *t*_(23)_ = −7.55, *p* < 0.001, *d*_*z*_ = −1.54.

#### Sustained N400 (500–700 ms)

The repeated measures omnibus-ANOVA revealed a tendency for a main effect of Condition, *F*_(1, 23)_ = 3.32, *p* = 0.081, η^2^_*p*_ = 0.126, a significant Laterality by Condition interaction, *F*_(2, 46)_ = 9.24, *p* < 0.001, ε = 0.947, η^2^_*p*_ = 0.502, a tendency for a Caudality by Condition interaction, *F*_(2, 46)_ = 2.96, *p* = 0.092, ε = 0.575, η^2^_*p*_ = 0.543. The three-way interaction between Laterality, Caudality, and Condition failed significance, *F*_(4, 92)_ = 1.86, *p* = 0.148, ε = 0.697, η^2^_*p*_ = 0.24.

Follow-up repeated measures ANOVAs for the three levels of Laterality separately revealed significant effects of Condition in Left, *F*_(1, 23)_ = 16.55, *p* < 0.001, η^2^_*p*_ = 0.418, and in Midline, *F*_(1, 23)_ = 14.49, *p* < 0.001, η^2^_*p*_ = 0.386, but no effect in Right, *F*_(1,23)_ < 1. The Condition effect was again strongest for the central-midline region around the Cz electrode, *t*_(23)_ = −4.42, *p* < 0.001, *d*_*z*_ = −0.9.

#### Late positivities (700–1000 ms)

The repeated measures omnibus-ANOVA revealed no significant main effect of Condition, *F*_(1, 23)_ = 1.22, *p* = 0.281, η^2^_*p*_ = 0.051. Condition significantly interacted with Laterality, *F*_(2, 46)_ = 5.72, *p* = 0.006, ε = 0.944, η^2^_*p*_ = 0.376, and Caudality, *F*_(2, 46)_ = 6.21, *p* = 0.017, ε = 0.554, η^2^_*p*_ = 0.382, whereas the three-way interaction failed significance, *F*_(4, 92)_ = 1.42, *p* = 0.234, ε = 0.718, η^2^_*p*_ = 0.132.

Follow-up repeated measures ANOVAs for each of the three levels of Laterality and Caudality separately revealed: a significant effect of Condition in Left, *F*_(1, 23)_ = 12.59, *p* = 0.002, η^2^_*p*_ = 0.353, no effect of Condition in Midline, *F*_(1, 23)_ = 2.24, *p* = 0.148, η^2^_*p*_ = 0.088, and a tendency for an effect of Condition in Right, *F*_(1, 23)_ = 3.85, *p* = 0.061, η^2^_*p*_ = 0.143. Further, a tendency for an effect of Condition in Anterior, *F*_(1, 23)_ = 4.89, *p* = 0.037, η^2^_*p*_ = 0.175, no effect in Central, *F*_(1, 23)_ = 2.32, *p* = 0.141, η^2^_*p*_ = 0.091, but a significant effect of Condition in Posterior, *F*_(1, 23)_ = 8.5, *p* = 0.007, η^2^_*p*_ = 0.269. Contrary to Experiment 2, the difference map at this time window revealed an enhanced posterior positivity that was slightly stronger at left-posterior electrodes (see Figure 6).

### Discussion

In line with our hypotheses, the main findings of this control experiment consisted of a long-lasting N400 and an enhanced late positivity to incoherent as compared to coherent endings. Whereas, the N400 effects showed high similarity to those obtained in Experiment 2, indicating their independence from experimental context, the late positivity clearly differed between both experiments. In Experiment 2, consisting of all three conditions, incoherent endings elicited an enhanced positivity over left anterior electrode sites rather than the expected posterior P600 component. The latter occurred to the same stimuli when joke endings were omitted from the experiment as realized here. This finding might indicate a strong sensitivity of the P600 to context variations, similar to other late ERP components. Schacht et al. (2014) have shown the P600 elicited by syntactic within-sentence violations in a sentence acceptability task to diminish below detectability when attention is directed away from the violations in a probe verification task. In Experiment 2 of the present study, the increased relevance of GP jokes compared to incoherent stimuli might have triggered similar mechanisms. Although judgments required were identical for participants in both experiments, the absence of joke endings in Experiment 4 might have implicitly increased (task) salience of incoherent endings compared to coherent endings. As already mentioned in the discussion in Experiment 3, also in Experiment 4 it is hard to tell how the decrease of one level in the experimental design might have interacted with possible repetition effects when comparing the results to Experiment 2.

## General discussion

GP jokes were described as a semantic-pragmatic phenomenon. They exploit a misleading discourse comprehension mechanism in order to amuse the recipient. The mental representation of the discourse based on the initially dominant interpretation is violated at the punch-line. Through a spreading activation of relevant semantic networks an alternative interpretation of the discourse is re-established. A successful belief revision in combination with the humorous content of the hidden interpretation is emotionally rewarded with mirth. Overall, our results from four experiments supported the hypothesized neuro-cognitive processes when compared to coherent texts on the one hand and to (discourse-)incoherent texts on the other hand: violation of the semantic representation, revision of the semantic representation, and emotional reaction.

The GP joke endings differed compared to the same texts with a coherent ending. They were harder to grasp, were rated as less predictable, triggered increased reading times for the final word due to the detection of the semantic violation and due to the revision of the semantic mental representation. Increased reading times for joke endings to coherent endings had previously been reported for similar stimulus material in English (Coulson et al., 2006). Further, the joke endings elicited the well-established N400 effect in the ERP data reflecting some (minor) difficulties at the stage of the semantic integration. The effect of increased reading times together with the appearance of the N400 effect, provides evidence for an automatic default interpretation of the ambiguous set-up. Participants committed to one dominant interpretation of the ambiguous textual input, rather than remaining undecided about the underspecified or misleading discourse. This initial incoherence led to the experience of incongruity which has been pointed out as a key element in the perception of humorous stimuli (e.g., Suls, 1972; Nerhardt, 1977; McGhee, 1979; Giora, 1991; Forabosco, 1992).

In the processing stages following the locus of the N400, we found mixed evidence that was strongly affected by the experimental contexts. In Experiment 2, joke endings also elicited a weak sustained N400 effect. In Experiment 3, however, where incoherent endings were omitted, this sustained N400 did not occur. Instead, a marginal negative component over left anterior electrode sites was elicited around 600 ms after the stimulus onset. This is in contrast with findings from studies by Coulson and Kutas (2001), and Coulson and Lovett (2004) who reported larger LLAN effects for better comprehenders but an enhanced N400 effect for poor comprehenders. According to the present findings, there is no reliable evidence for this component. If there is a LLAN component that truly reflects additional processing effort during discourse comprehension, or in particular the semantic re-interpretation necessary for the re-establishment of a coherent discourse during joke comprehension, then this component is characterized by three points: It is (i) weak in effect size, hence not reliably elicited in experiments; (ii) strongly susceptible to individual discourse comprehension ability; and (iii) strongly susceptible to contextual influences that disable participants to engage in additional processing effort for successful comprehension.

Contrary to previous findings with joke material (Coulson and Lovett, 2004; Marinkovic et al., 2011), no evidence was found for the hypothesized (posterior) P600 component, which had also been argued to be related to the re-establishment of coherence. We could not find any hint for a similarity between the well-studied (mainly syntactic) GP sentences and the (mainly semantic) GP jokes. Nevertheless, we did find a frontal late positivity for the joke endings in Experiment 2 which was diminished and non-significant in Experiment 3. It appeared to be comparable to the frontal P600 which had been found for ambiguous and complex syntactic sentence parsing (Kaan and Swaab, 2003). It also was similar to a component reported for jokes that involved frame-shifting. In these findings, the component was mainly present for right-handed women (Coulson and Lovett, 2004). It is plausible that this frontal positivity does not reflect a cognitive processing stage, but might be related to the emotional reaction to the joke endings (see below).

As indicated by our data from ratings of humorous potential and from pupillometric recordings, joke comprehension was emotionally rewarded with the experience of mirth. Pupil diameter data on a stimulus level for joke endings were only correlated with the ratings of the humor potential of the joke. Furthermore, pupil diameters were significantly larger for the joke endings, but not for the incoherent endings. Since incoherent endings were also assumed to increase cognitive load during comprehension, as indicated by increased reading times in Experiment 1, we propose that the differences between pupil diameters for joke compared to incoherent and coherent endings reflect the emotional reaction of mirth, similarly to the enhanced mean ERP amplitudes of the late positivity at prefrontal electrode sites in Experiment 2. This assumption is supported by similar ERP findings in a previous study by Du and co-workers (Du et al., 2012), investigating verbal humor in the Chinese language. However, late ERP positivities that have been related to either emotional stimulus processing (e.g., Schacht and Sommer, 2009b; Bayer et al., 2012) or to emotional responses in humor processing (e.g., Gierych et al., 2005; Korb et al., 2012) typically show a more posterior distribution over the scalp surface (e.g., Gierych et al., 2005; Korb et al., 2012). Even though we controlled for humorous potential of our stimulus material, it was economically not feasible to collect individual ratings of the subjective experience of mirth for the participants of this study. Relating such idiosyncratic ratings to the individual amplitudes of the frontal positivity component could help to uncover the functional significance of late positivities in different kinds of emotional processing.

In the present study, we not only compared the processing of joke endings with coherent but also with incoherent text endings. Incoherent endings were harder (or impossible) to be semantically integrated in the discourse. This was reflected by longer reading times compared to the coherent endings and mainly by stronger and prolonged N400 amplitudes compared to both the joke and the coherent endings. Further, the incoherent endings elicited late positivities, which scalp distribution interestingly showed strong context sensitivity. They were also accompanied with reduced pupil diameters, probably related to the absence of an emotional response. The differences in the N400 component suggest that the processing of totally incoherent endings starts to diverge from the processing of the joke endings at an early stage of discourse comprehension. While the joke endings very early seemed to trigger the search for the alternative interpretation and the revision of the semantic representation, as suggested by the reduced N400 effect, this process appears to be absent for the incoherent endings. Following processing stages of incoherent endings were strongly modulated by the context. The mere presence of the joke endings in Experiment 2 seemed to have altered the processing after the detection of the semantic incoherence compared to the processing of the very same endings in Experiment 4. In the latter experiment, incoherent endings elicited a posterior P600 activation. One possible explanation could be that the absence of joke endings made it clear to the participants that no coherent interpretation was provided. Accordingly, participants were better able to engage in monitoring strategies that signal the impossibility of a successful comprehension. A similar functional equivalent of the P600 component had previously been proposed by Van Herten et al. (2005).

Limitations of the study have to be pointed out. The whole lab situation is an artificial setting. A large amount of short texts had to be read on a computer screen in a way, which might be very distinct from natural reading processes (moving windows paradigm, RSVP), and especially from the usual social interaction of joke telling. Furthermore, participants read every text fragment three times or two times respectively in the control studies. Even though we balanced out the order of the conditions for the participants, this repetition probably had an impact on the processing of all three conditions. However, this design had the advantage of keeping all experimental settings but the final word between the three conditions completely identical.

Further research should address more precisely the functional relationships of the described ERP components. Especially the interpretation of the frontal positivity for joke endings compared to both to coherent and to incoherent endings, found in Experiment 2 and in Experiment 3, remains speculative. Since the processing of joke endings in the present study is assumed to differ from the other conditions by (at least) two features, the revision and the emotional response, this component could be related to any of these two processes. Siebörger (2006) carried out an fMRI study in order to disentangle the cognitive processes of joke comprehension from the emotional reactions. He compared GP jokes (revision plus emotional reaction) to texts which he called “Revisionsgeschichten (revision texts)” (revision but no emotional reaction), to straight coherent (no revision needed) and to incoherent texts (no coherence, no revision, no emotional reaction). The results indicated differences for all three hypothesized processes. A similar design as an ERP-study would be a very useful next step. Future research could also investigate the nature of the N400 during joke comprehension in more detail by manipulating the salience of the initially dominant interpretation (possible increase of the N400 effect) and the accessibility of the hidden interpretation (possible increase of the LLAN component or reduction of the N400 effect) of the joke endings by contextual priming prior to the stimulus presentation (cf. Mayerhofer and Schacht, 2013).

The present experiments do not allow distinguishing the theoretical account of frame-shifting (Coulson and Kutas, 1998; Coulson, 2001) from the focus on the revision, as in the present account. Both accounts share the general idea of a re-interpretation process that is additionally supported by the present findings. This re-interpretation is in line with the assumption of incremental and non-monotonic reasoning processes based on inferential belief updates and permanent and active constructions of situation models during language comprehension aiming to represent the meaning of a text (Baggio et al., 2008). Even if an interpretation once has been established, this interpretation can be overridden by an alternative explanation which is better able to integrate the new textual evidence.

### Conflict of interest statement

The authors declare that the research was conducted in the absence of any commercial or financial relationships that could be construed as a potential conflict of interest.

## Footnotes

^1^Attributed to Bob Monkhouse, retrieved from http://www.bitcomedy.co.uk/comedy-features/101-short-jokes/ on the 4th of June, 2013.

^2^Additional factors probably render incoherence incongruous in the sense of humor theories. For the current purpose, we will use the term incoherence when we refer to discourse comprehension and the term incongruity when it is related to humor theory.

^3^Since previous ERP studies on joke processing used paired common references located at or closed to the mastoids, we re-calculated our data to both average reference (AR; averaged across all electrodes) and average mastoid reference (MR) averaged across the left and right mastoids. Reports on statistics are limited to AR data. However, in order to allow for comparisons between previous and the present findings, we will follow the recommendations by Picton et al. (2000) and depict both AR and MR data in separate panels (cf. the legend of Figure 3).
