# Supplementary material for: From incoherence to mirth: neuro-cognitive processing of garden-path jokes
Source: Front Psychol. 2015 May 12;6:550. doi: 10.3389/fpsyg.2015.00550 (PMC4429229; doi:10.3389/fpsyg.2015.00550)
Supplement: Supplementary file 1 [file Table1.PDF]

# Supplementary Material

# From incoherence to mirth: neuro-cognitive processing of garden-path jokes

**Bastian Mayerhofer1[1]*, Annekathrin Schacht2[1]**

[1]CRC Text Structures, University of Göttingen, Göttingen, Germany

*** Correspondence:** Bastian Mayerhofer, CRC Text Structures, University of Göttingen, Nikolausberger Weg 23, 37073 Göttingen, Germany.
E-mail: bmayerh@gwdg.de

## Supplementary Data

Stimulus material was taken and adapted from internet collections (retrieved from http:// www.ahajokes.com/, http://www.jokedatabase.org, www.dein-witz.de, witze -datenbank.de, www.witze-ohne-ende.de, http://www.brainyquote.com/quotes/ authors/s/steven_wright.html, in August, 2011), from various books and previous articles on verbal humor (Attardo & Raskin, 1991; Coulson & Kutas, 1998, 2001; Coulson, 2001; Coulson & Lovett, 2004; Dynel, 2009; Ritchie, 2001), from personal memory, and from friends.

- Mami, Mami, ich bin jetzt 14 Jahre alt. Darf ich endlich einen BH und Make-up tragen? Nein, und iss deine Suppe auf, mein Junge/Mädchen/Onkel. *(Mummy, mummy, I am fourteen years old now. Can I finally wear a bra and make-up? No, and now eat up your soup, my son/girl/uncle.)*
- Der Polizist hält eine Autofahrerin an und ermahnt sie: Sie können doch nicht mit 80 durch das Ortsgebiet fahren. Darauf erwidert die Dame: 80? Ich muss wohl mal wieder liften/bremsen/husten. *(The policeman stops a female driver and warns her: You must not drive through an urban area with 80. The lady answers: 80? Looks like it's time for another facelift/to break/to cough!)*
- Der Patient klingelt beim Haus seines Arztes. Die Frau des Arztes öffnet die Tür. Mit kranker Stimme flüstert der Patient: Ist der Arzt da? Die Frau flüstert zurück: Nein, kommen Sie herein/ morgen/daneben! *(The patient rings at the door of his doctor's home. The doctor's wife opens the door. In a husky voice he whispers: Is the doctor home? The wife replies: No, please come in/ tomorrow/next door.)*
- Eine Frau fragt die Verkäuferin im Kleiderladen: Dürfte ich das Kleid im Schaufenster anprobieren? Die Verkäuferin antwortet: Ja, aber besser wäre die Kabine/Bluse/Soße. *(A woman asks the seller in a clothing store: May I try on the dress in the shop window? The seller answers: Yes, but it would be better to use the fitting room/blouse/sauce*.)
- Ein Freund zu seinem Freund, der gerade aus einer Wellness-Therme kommt: Hast du ein Bad genommen? Darauf der andere: Nein, nur Handtücher/Massagen/Kaninchen. *(A friend says to his friend, who just comes out of a spa: Did you take a bath? The other one replies: No, only some towels/massages/rabbits.)*
- Ich lasse meinen Steuerberater meine Buchhaltung durchführen. Das spart Zeit. Letztes Jahr ersparte ich mir damit zehn Jahre/Tage/Schuhe. (*My accountant does my bookkeeping. This saves me time. Last year, it saved me 10 years/days/shoes.*)

- Ein Mann zum Barkeeper: Können Sie mir etwas empfehlen, das kalt ist und voller Rum? Der Barkeeper antwortet: Ja, ich empfehle Ihnen meine Frau/Drinks/Musik. *(A man to a bartender: Can you recommend me something that is cold and full of rum? The bartender replies: Yes, I recommend my wife/drinks/music.)*
- Eine neue Studie untersuchte die Schlafgewohnheiten von Studenten. Der durchschnittliche Student steht um halb 8 Uhr in der Früh auf. Dann geht er schlafen/lernen/werfen. (*A new study examines the sleeping habits of college students. The average college student gets up at 8am. Then he goes to sleep/study/throw.*)
- Eine neue Studie besagt, dass in Deutschland pro Jahr 720 Mio. Euro für Glücksspiel ausgegeben werden. Gut 50 Prozent davon entfallen auf Heiraten/Pokern/Forschen. (*A new study shows that 720 million Euros are spent on gambling each year in Germany. 50 percent of it is based on marriages/poker/research.*)
- Meine Freundin hat so viel Negatives über Rauchen gelesen. Deshalb hat sie beschlossen, aufzuhören, mit dem Lesen/Rauchen/Reden. (*My girlfriend has read many negative things about smoking. Therefore she decided to quit reading/smoking/talking*.)
- Ein Bauer erzählt einem anderen Bauern: Gestern habe ich den Hof gegen Feuer und Hagel versichert. Darauf der andere Bauer: Gegen Feuer, das finde ich gut. Aber Hagel kann man nicht erzeugen/versichern/bedienen. (*A farmer tells another farmer: Yesterday I insured my farm against fire and hail. The other farmer replies: Against fire, I like that. But hail you cannot produce/ensure/serve!*)
- Ich wurde aus dem Kino geworfen, weil ich mein eigenes Essen mitbrachte. Aber die Preise für die Snacks im Kino sind so unverschämt hoch. Außerdem hatte ich Lust auf Grillen/Sandwiches/Badminton. (*I was kicked out of the cinema because I brought my own food. But the prices for snacks in cinemas are off the roof. Also, I felt like having a BBQ/sandwich/ (playing) badminton.*)
- Immer wenn ich Sie lachen sehe, Frau Müller, möchte ich zu ihnen sagen: Bitte kommen Sie doch mal zu mir. Na, na. Sie sind mir vielleicht ein Casanova! Nein, ich bin ein Zahnarzt/Liebhaber/Schriftsteller. (*Everytime I see you laugh, Misses Müller, I want to say to you: Please, come see me soon. Now, now, you are a Casanova! No, I am a dentist/lover/writer.*)
- Der Klassenlehrer zur stolzen Mutter: Ihr Sohn ist außerordentlich kreativ und voller neuer Ideen. Vor allem beim Rechtschreiben/Musizieren/Produzieren. (*The teacher says to the proud mother: Your son is extremely creative and full of new ideas. Especially when it comes to spelling/music/producing.*)
- Die Frau zieht ihren Mann in das Schlafzimmer und sagt zu ihm: So, bitte zieh meine Bluse, meinen BH und meine Unterhose aus. Ich will nicht, dass du meine Sachen anprobierst/zerreißt/verkaufst. (*The wife pulls her husband into the bedroom and says: Alright, now please take of my blouse, my bra and my panties. I don’t want you to try/destroy/sell my clothes.*)
- Ein Freund beschwert sich bei einem anderen: Meine Freundin meint, ich sei viel zu neugierig. Zumindest schreibt sie das in ihrem Tagebuch/Reisebericht/Scheckbuch. (*A friend complains to his friend: My girlfriend says I am way too curious. At least, that’s what she wrote in her diary/journey report/cheque book.*)
- Eine Frau sagt zu ihrer Freundin: Mein Mann ist ein Engel. Die andere antwortet: Da hast du aber mehr Glück als ich, weil meiner noch lebt/trinkt/schläft. (*A woman says to her girlfriend: My husband is an angel. The other woman replies: You are luckier than I am, because my husband is still alive/drinking/asleep.*)
- Der Arzt sagt zum Patienten: Ich kann die Ursache ihrer Beschwerden nicht entdecken. Ehrlich gesagt, glaube ich, das kommt vom Trinken. Der Patient antwortet: Gut, ich komme in einer Woche wieder. Vielleicht sind Sie dann nüchtern/schlauer/verwaist. (*The doctor says to his patient: I cannot detect the cause of your medical condition. To be honest, I think it’s because of the drinking.*

*The patient replies: Fine, I will come back in a week. Maybe by then you will be sober/smarter/ orphaned.*)

- Sag mal, Susi. Warum fütterst du deinen Kater mit Sonnenblumenkernen? Susi antwortet: Sonnenblumenkerne sind das Lieblingsfutter von meinem Vogel/Kater/Affen. (*Tell me, Susi. Why do you feed sunflower seeds to your cat? Susi replies: Sunflower seeds are my bird's/cat's/monkey's favorite food.*)
- Eine Kuh sagt zu der anderen: Fürchtest du dich eigentlich vor dem Rinderwahn? Darauf die andere: Wieso? Ich bin doch ein Flugzeug/Hausrind/Enkelkind! (*A cow says to another: Are you afraid of the mad cow desease? The second cow replies: Why would I? I am an airplane/hose cow/ nephew!*)
- Der Schwimmer fragt den Lifeguard: Und sind Sie auch sicher, dass hier keine Haigefahr herrscht? Der Lifeguard beruhigt den Schwimmer. Ja, ganz sicher. Die vertragen sich nicht mit den Krokodilen/Algenarten/Ergebnissen. (*The swimmer asks the life guard: And you are sure that there is no danger of shark attacks here? The life guard calms the simmer. Yes, absolutely sure. They don't get along with the crocodiles/algae kinds/results.*)
- Der Ehemann zu seiner Frau: Schatz, ich habe einen tollen Job gefunden. Die Frau findet das wunderbar. Darauf sagt der Mann: Toll, morgen ist der erste Tag für dich/mich/ihn. (*The husband to his wife: Darling, I found a great job. The wife is delighted. Thereupon the husband says: Great, tomorrow is your/my/his first day.*)
- Was ist denn mit dir los? fragt der Wirt den unglücklichen Stammgast. Ach, meine Frau ist mit meinem besten Freund durchgebrannt. Alles ist so sinnlos ohne Freund/Frau/Ball. (*What's the matter with you?, the host asks an unhappy regular guest. Oh well, my wife ran away with my best mate. Everything is meaningless without my mate/wife/ball.*)
- Ich würde gerne so wie mein Großvater, sanft und ruhig im Schlaf sterben. Nicht angsterfüllt und schreiend wie seine Fahrgäste/Kameraden/Vertreter. (*I want to die like my grandfather, calm and peaceful during sleep. Not frightened and screaming like his passengers/comrades/assistants.)*
- Ein Blinder kommt mit seinem Hund an der Leine in eine Bar. Plötzlich schwingt er den Hund über seinen Kopf mit der Leine im Kreis. Der Barkeeper schreit ihn an: Was machen Sie da? Der Blinde antwortet: Ach, einfach nur herumschauen/herumschwingen/herumschwimmen. (*A blind man and his dog walk into a bar. Suddenly he spins the dog on his leash above his head. The bartender yells: What are you doing? The blind man replies: Well, just looking/swinging/swimming around.*)
- Die alte Hebamme kommt völlig aufgelöst zum Pfarrer. Mein Mann ist gestern verstorben. Der Pfarrer versucht sie zu trösten. Das ist ja furchtbar. Hatte er einen letzten Wunsch? Sie antwortet: Ja, seine letzten Worte waren: Bitte nicht schießen/weinen/hören! (*The old midwife comes up to the pastor with despair. My husband died yesterday. The pastor tries to comfort her. That is awful. Did he have a last wish? She replies: Yes, his last words were: Please don't shoot/cry/listen!*)
- Der Sohn eines Rechtsanwalts fragt seinen Vater: Sagt ein Rechtsanwalt jemals die Wahrheit? Der Vater antwortet: Ja, ein Rechtsanwalt macht alles, was hilft, um einen Fall zu gewinnen/klären/ teilen. (*The son of a lawyer asks his father: Does a lawyer ever say the truth? The father replies: Yes, a lawyer does whatever it takes to win/solve/share a case.*)
- Ein Rechtsanwalt verteidigt den Angeklagten, der seine beiden Eltern ermordete. Ich plädiere auf mildernde Umstände für meinen Klienten. Bedenken Sie, er ist ein Waisenkind/Geisteskranker/ Kartenleger. (*A lawyer represents a defendant who killed his parents. I plead in favor of mitigating circumstances for my client. Please consider that he is a/an orphan/lunatic/???*)
- Ein Arzt hatte Sex mit einem seiner Patienten. Er berichtet es seinem Freund und rechtfertigt sich bei ihm: Ich bin sicher, das machen viele andere Ärzte auch. Der Freund antwortet: Ja, aber ich denke, von denen bist du der einzige Tierarzt/Frauenarzt/Malermeister. (*A doctor had sex with one*

*of his patients. He tells it to his friend and justifies himself: I am sure, many doctors do this. His friend replies: Yes, but I think you are the only one among them who is a vet/gynecologist/painter!*)

- Sie hat ihr gutes Aussehen von ihrer Mutter. Die Mutter ist Schönheitschirurgin/ Unterwäschenmodel/Drogenkommissarin. (*She has got her beauty from her mother. The mother is a plastic surgeon/underwear model/drug police.*)
- Ich leide nicht mehr unter Geisteskrankheit. Jeden einzelnen Augenblick meines Lebens kann ich die Krankheit genießen/kontrollieren/vorbereiten. (*I no longer suffer from mental disease. Every moment of my life I can enjoy/control/prepare my illness.*)
- Ich habe zwei Warnschüsse abgefeuert. Beide in den Kopf/Himmel/Ofen. (*I fired two warning shots. Both in the head/sky/oven*)
- Letztens habe ich mit ansehen müssen, wie sechs Männer meinen Chef verprügelten. Ich fühlte mich schlecht, weil ich nicht geholfen habe. Aber sechs Männer sind echt genug/zuviel/anders. (*Recently, I had to watch how my boss was beaten up by six men. I felt guilty, because I didn't help. But six men were simply enough/too much/too different.*)
- Ich habe noch immer den Körper einer 18-Jährigen. Und zwar in meinem Keller/Alter/Bild. (*I still have the body of an eighteen-year-old. It is in my cellar/age*/picture.*)
- Dreißig Jahre lang waren meine Frau und ich glücklich und zufrieden. Dann haben wir uns getroffen/getrennt/beliefert. (*For thirty years my wife and I were happy and content. Then we met/ separated/delivered.*)
- Über vierzig Jahre habe ich nun nur eine Frau geliebt. Ich hoffe, dass meine Frau das nie erfährt/ vergisst/mietet. (*For more than forty years I have only loved one woman. I hope, my wife will never know/forget/rent.*)
- Ich bin so stolz. Viermal die Woche gehe ich ins Fitness-Center. Bald mache ich dort auch einen Work-Out/Yoga-Kurs/Surf-Kurs. (*I am so proud. I go to the gym four times a week. Soon I will do a workout/yoga-class/surf school.*)
- Ich hasse es, wenn Leute Freddie Mercury mit Gott vergleichen. Ich meine, er ist gut. Aber doch nicht so gut, wie der Rockstar/Allmächtige/Zirkusdirektor. (*I hate it when people compare Freddie Mercury with God. I mean, he's good. But he's not as good as the rock star./almighty/circus director.*)
- Die OP-Assistentin warnt den Chirurgen: Doktor, schneiden Sie nicht zu tief. Sie beschädigen sonst noch den OP-Tisch/Herzmuskel/Autolack. (*The surgical nurse warns the surgeon: Doctor, don't cut too deep. Otherwise you will damage the surgical table/cordial muscle/car paint.*)
- In New York wird alle zwei Minuten eine Person von einem Auto überfahren. Das ist eine große gesundheitliche Gefahr für die Person/Bevölkerung/Polizei. (*Every other minute a person gets run over by a car in New York. This is a great health risk for the person/population/police.*)
- Ich habe den Wien-Marathon in unter drei Stunden beendet. Ich wäre eigentlich noch schneller gewesen. Aber ich musste eine längere Pause machen, um zu tanken/trinken/tanzen. (*I finished the Vienna Marathon in less than four hours. In fact, I could have been even faster. But I had to take a long break to refuel/drink/dance.*)
- Ich bin kürzlich auf einem Pferd geritten. Der hintere Fuß des Pferdes war so stark beeinträchtigt, dass ich es leider erschießen musste. Alle waren total darüber entsetzt, also alle anderen in dem Kinderkarussell/Reitstall/Turnvererin. (*Lately I rode a horse. His hind hoof was extremely damaged so that I had to shoot it. Everyone was completely shocked about this, I mean everyone in the children's roundabout/riding stall/gym.*)
- Um vier Uhr in der Früh ging heute der Autoalarm von meinem Nachbarn los. Ich bin so furchtbar erschrocken, dass ich sofort weglief/aufwachte/aufaß. (*At 4 am this morning the alarm of my neighbor's car went off. I was so startled, I immediately ran away/woke up/ate up.*)

- Glaub mir! Dir wehzutun ist das Allerletzte, was ich vorhabe. Aber es ist eingeplant/unvermeidbar/unberührbar. (*Believe me! The last thing I intend to do is to hurt you. But it is scheduled/unavoidable/untouchable.*)
- Einmal pro Woche gehen meine Frau und ich in ein schickes Restaurant. Danach schauen wir einen romantischen Film im Kino und danach haben wir leidenschaftlichen Sex. Manchmal machen wir das auch miteinander/mehrmals/minütlich. (*Once a week my wife and I go to a fancy restaurant. Afterwards we watch a romantic movie at the cinema and then we have passionate sex. Sometimes we even do it together/multiple times/every minute.*)
